# Arbuscular Mycorrhizal Fungal 14-3-3 Proteins Are Involved in Arbuscule Formation and Responses to Abiotic Stresses During AM Symbiosis

**DOI:** 10.3389/fmicb.2018.00091

**Published:** 2018-03-05

**Authors:** Zhongfeng Sun, Jiabin Song, Xi’an Xin, Xianan Xie, Bin Zhao

**Affiliations:** ^1^State Key Laboratory of Agricultural Microbiology, College of Life Science and Technology, Huazhong Agricultural University, Wuhan, China; ^2^State Key Laboratory for Conservation and Utilization of Subtropical Agro-Bioresources, College of Forestry and Landscape Architecture, South China Agricultural University, Guangzhou, China

**Keywords:** arbuscular mycorrhiza, abiotic stresses, *Funneliformis mosseae*, *Rhizophagus irregularis*, *Fm201*, host-induced gene silencing, 14-3-3 proteins

## Abstract

Arbuscular mycorrhizal (AM) fungi are soil-borne fungi belonging to the ancient phylum Glomeromycota and are important symbionts of the arbuscular mycorrhiza, enhancing plant nutrient acquisition and resistance to various abiotic stresses. In contrast to their significant physiological implications, the molecular basis involved is poorly understood, largely due to their obligate biotrophism and complicated genetics. Here, we identify and characterize three genes termed *Fm201*, *Ri14-3-3* and *RiBMH2* that encode 14-3-3-like proteins in the AM fungi *Funneliformis mosseae* and *Rhizophagus irregularis*, respectively. The transcriptional levels of *Fm201*, *Ri14-3-3* and *RiBMH2* are strongly induced in the pre-symbiotic and symbiotic phases, including germinating spores, intraradical hyphae- and arbuscules-enriched roots. To functionally characterize the *Fm201*, *Ri14-3-3* and *RiBMH2* genes, we took advantage of a yeast heterologous system owing to the lack of AM fungal transformation systems. Our data suggest that all three genes can restore the lethal *Saccharomyces cerevisiae bmh1 bmh2* double mutant on galactose-containing media. Importantly, yeast one-hybrid analysis suggests that the transcription factor RiMsn2 is able to recognize the STRE (CCCCT/AGGGG) element present in the promoter region of *Fm201* gene. More importantly, Host-Induced Gene Silencing of both *Ri14-3-3* and *RiBMH2* in *Rhizophagus irregularis* impairs the arbuscule formation in AM symbiosis and inhibits the expression of symbiotic *PT4* and *MST2* genes from plant and fungal partners, respectively. We further subjected the AM fungus-*Medicago truncatula* association system to drought or salinity stress. Accordingly, the expression profiles in both mycorrhizal roots and extraradical hyphae reveal that these three 14-3-3-like genes are involved in response to drought or salinity stress. Collectively, our results provide new insights into molecular functions of the AM fungal 14-3-3 proteins in abiotic stress responses and arbuscule formation during AM symbiosis.

## Introduction

Arbuscular mycorrhizal (AM) fungi, belonging to the ancient phylum Glomeromycota, are soil-borne microbes and capable of establishing the most widespread mutualistic association, namely AM symbiosis, with more than 80% terrestrial flowering plant species (Simon et al., 1993; Remy et al., 1994). Due to the obligate biotrophic nature, AM fungi need to consume plant photosynthates (Bago et al., 2000) and lipids to complete their life cycle (Bravo et al., 2017; Jiang et al., 2017), and reciprocally AM fungi significantly contribute to plant growth not only by enhancing mineral nutrient uptake and water acquisition from surrounding soil, but also protecting plants against fungal pathogens (Smith and Read, 2008; Jung et al., 2012; Chitarra et al., 2016) and a variety of abiotic stresses (Augé, 2001; Schützendübel and Polle, 2002; Lenoir et al., 2016). Therefore, AM fungi are key endosymbionts of the plant symbiosis and have significant impacts on plant productivity and ecosystem function (Van der Heijden et al., 1998), and are of great interest for the sustainable agricultural development (Gianinazzi et al., 2010).

The formation of a functional AM symbiosis requires successive stages between AM fungal and host symbionts at both physiological and molecular levels (Genre et al., 2005; Bonfante and Genre, 2010). Specifically, the development of arbuscular mycorrhiza consists of three major distinct stages through the progression of AM fungal hyphae during root colonization (Genre et al., 2005; Harrison, 2012; Gutjahr and Parniske, 2013). Arbuscules are generally thought to be the primary sites for nutrients exchange between the two symbionts (Parniske, 2008; Bonfante and Genre, 2010). In this symbiotic interface, the host membrane surrounding an arbuscule, known as the periarbuscular membrane (PAM), harbors AM-specific Pi transporters that acquire Pi released from the arbuscule (Harrison et al., 2002; Javot et al., 2007a). Outside the roots, the extraradical mycelia of AM fungi can extend the soil substratum beyond the depletion zone of the rhizosphere to uptake nutrients (particularly Pi and N) and water from the surrounding soils (Govindarajulu et al., 2005; Javot et al., 2007b; Li et al., 2013).

Despite their great importance, the underlying signaling events during initiation and formation of AM symbiosis are not well understood (Paszkowski, 2006; Bonfante and Requena, 2011; Gutjahr and Parniske, 2013; Oldroyd, 2013; Schmitz and Harrison, 2014; Bonfante and Genre, 2015). In contrast to a plethora of discoveries on morphological and chemical features in AM fungi, the molecular basis involved is still largely unknown, partially due to the limited available genomic resources. Many genome-wide gene expression analysis have been employed recently in order to understand the underlying molecular mechanisms of the AM formation. These studies mainly focused on the host plants (recently reviewed in Salvioli and Bonfante, 2013), whereas only a few investigations addressed the fungi partners (Requena et al., 2002; Breuninger and Requena, 2004; Cappellazzo et al., 2007; Kikuchi et al., 2014). Major progress has been recently achieved using transcriptomics and genomics data of *Rhizophagus irregularis* (Tisserant et al., 2012; Tisserant et al., 2013; Lin et al., 2014) and *Gigaspora* genus (Salvioli et al., 2016; Tang et al., 2016).

Using the suppression subtractive hybridization library (SSH) strategy, Breuninger and Requena (2004) firstly found some ESTs of fungal genes which were induced in the appressorium stage may display potential roles in this stage of *Funneliformis mosseae*. In this case, an EST tag termed 201, which encodes a 14-3-3 like protein in fungi, shows a significant up-regulation in the appressorium stage of AM symbiosis (Breuninger and Requena, 2004). Recently, Tisserant et al. (2012) released the first genome-wide overview of the transcriptional profiles of the various fungal tissues of *R. irregularis*. Particularly, a large number of fungal non-redundantly expressed transcripts was investigated in spores, intraradical mycelia (IRM), extraradical mycelia (ERM), and arbuscules. Interestingly, the transcripts encoding *R. irregularis* 14-3-3 proteins were inducible in both IRM and ERM.

14-3-3 proteins are highly conserved and dimeric proteins with a subunit mass of approximate 30 KDa (van Heusden and Steensma, 2006). These proteins are named based on the fraction number after EDTA-cellulose chromatography and the position after subsequent starch gel-electrophoresis (Moore, 1967). The first description of the function of 14-3-3 protein is substantially comparable to the ‘activator’ protein, that is important in the regulation of serotonin and noradrenaline biosynthesis in the brain (Ichimura et al., 1987). Moreover, 14-3-3 proteins form homo- or hetero-dimers by two subunits harboring the independent ligand-binding channels. Until now, it is extensively studied that these proteins generally serve as adapters, chaperones, activators, or repressors in the regulation of signal transduction pathways by reorganization of specific phosphoserine/phosphothreonine-inclusive binding motifs phosphorylated by protein kinase A (Smith et al., 1998; van Heusden, 2009; Smith et al., 2011; Parua and Young, 2014). Additionally, 14-3-3 proteins also play important roles in the pseudohyphal growth of *Saccharomyces cerevisiae* and the pathogenic fungal infection, such as *Ustilago maydis* (Gancedo, 2001; Rispail et al., 2009; Ballou et al., 2013; Liu et al., 2015). These known 14-3-3 proteins have also been implicated in several signaling cascades responding to biotic and abiotic stresses in plants (Roberts et al., 2002; Lozano-Duran and Robatzek, 2015; Li et al., 2016), suggesting that these proteins may display distinct roles during eukaryotes life cycle (Liu et al., 2015). So far, at least two distinct 14-3-3 subunits have been characterized in fungi (Darling et al., 2005; Hermeking and Benzinger, 2006). Porcel et al. (2006) identified a gene *Gi14-3-3* (currently *Ri14-3-3*) from the AM fungus *R. irregularis*, encoding a 14-3-3 protein subunit that is enhanced under drought stress during AM symbiosis, being the first 14-3-3 protein from AM fungus reported so far. Additionally, recent work has provided new evidence for the potential involvement of *Ri14-3-3* gene in the interaction between maize and *R. irregularis* under drought stress (Li et al., 2016). However, the molecular mechanisms of *Ri14-3-3* gene in enhancing plant resistance to drought stress are still unclear.

To further advance our understanding of the roles of 14-3-3 proteins in fungal symbionts during AM symbiosis, we here report three novel fungal genes, so called *Fm201*, *Ri14-3-3* and *RiBMH2*, which encode 14-3-3-like proteins from *F. mosseae* (BEG12) and *R. irregularis* (DAOM197198), respectively. *14-3-3* genes are strongly induced in the early stage of AM symbiosis. Moreover, the expression of *14-3-3* genes are regulated in response to drought and osmotic stresses. To further characterize these AM fungal *14-3-3* genes, we validated the capability of these genes to complement the metabolic deficient Δ*bmhs* mutant (*bmh1* and *bmh2* double mutant) in a yeast heterologous expression system. We also provided insights into the regulatory mechanism between 14-3-3 protein and Msn2 transcription factor from AM fungi and further proved the existence of two distinct 14-3-3 subunits in AM fungi. More importantly, in the absence of stable transformation protocols for AM fungi (Helber and Requena, 2008; Helber et al., 2011), host-induced gene silencing (HIGS) of the two *14-3-3* genes in *R. irregularis*, whereby these genes are silenced in the AM fungal symbiont by expressing an RNA interference construct in the host, provides a potential tool to address the function of 14-3-3 proteins in obligate biotrophic AM fungi. Collectively, our results provide new insights into molecular functions of the AM fungal 14-3-3 proteins in stress responses and arbuscule formation during AM symbiosis.

## Results

### Identification of *Fm201* Gene From *Funneliformis mosseae*

In the previous study, transcript abundance of 201-tag was significantly enhanced at the early appressorium stage of AM symbiosis (Breuninger and Requena, 2004). The amino acid sequence of 201-tag exhibits a high similarity (∼97%) with the Ri14-3-3 protein from *R. irregularis* (Breuninger and Requena, 2004; Tisserant et al., 2012). With the aim to confirm if this fungal 14-3-3 protein is involved in AM symbiosis, a DNA clone of 1.5 kb in length was obtained by inverse PCR from the cloning procedures on *F. mosseae* genomic DNA based on the 201-tag. The isolated fragment with 5′ end and upstream region was highly similar to the sequence of *Ri14-3-3* gene from *R. irregularis* (Porcel et al., 2006). Since the 5′ and 3′ end sequences of this gene are not available, 5′ and 3′ RACE experiments on RNA pools of *F. mosseae* germinating spores were subsequently performed to obtain the full-length CDS sequence. A 1,401 bp full-length cDNA sequence of *Fm201*, covering the 5′UTR (188 bp) and 3′UTR (411 bp), was thus identified (Accession number: KM258580). The corresponding genomic sequence of *Fm201* gene is 1,685 bp in length, containing seven exons and seven introns (Supplementary Figure S1). Interestingly, *Fm201* gene contains a 100 nt intron in the 5′ UTR and two transcriptional variants of 3′UTRs (61 and 411 nt in length, respectively). These unusual features of AM fungal 14-3-3 gene firstly reported in the present study may suggest important roles in the regulation of *Fm201* expression during AM symbiosis.

### Fm201 Protein Is Conserved Among Eukaryotes

**FIGURE 1:**
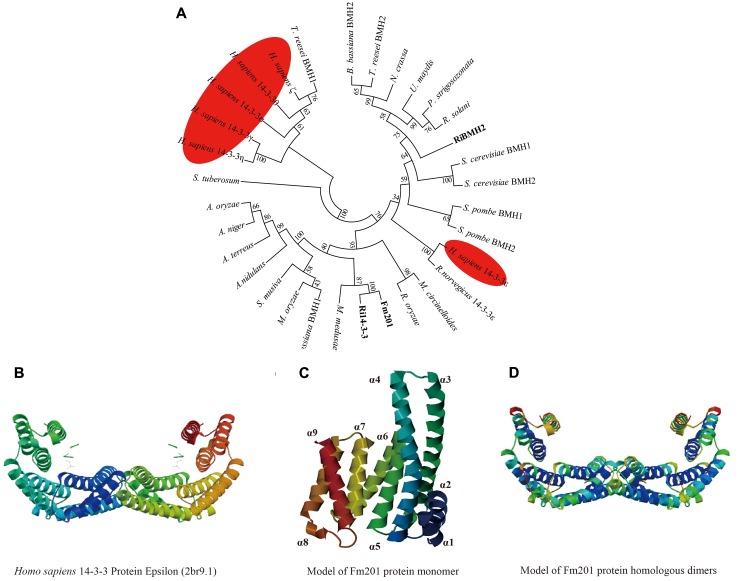
*In silico* analysis of Fm201 protein and its homologs from AM fungi. **(A)** The unrooted phylogenetic tree was constructed based on multiple sequence alignment of 14-3-3 proteins in fungal species and *Homo sapiens*. The phylogenetic relationships were analyzed by Neighbor-joining method with MEGA v6.0 software. Bootstrap values were calculated using 1,000 replicates. 14-3-3 proteins from mycorrhizal fungi are highlighted in bold and their homologs from *H. sapiens* are colored red. **(B)** Predicted structure of *Homo sapiens* 14-3-3 epsilon (2br9A) dimers. **(C,D)** Homology modeling of Fm201 monomer and homodimer using *Homo sapiens* 14-3-3 epsilon (2br9A) as reference.

To further investigate the phylogenetic and structural features of the Fm201 protein from AM fungi, we exploited the phylogenetic placement and 3D structure of Fm201 protein using bioinformatics strategy. The *in silico* analysis revealed that the open reading frame (ORF) of *Fm201* gene consists of 804 bp corresponding to 267 amino acids with a predicted molecular weight of approximate 30 kDa. A phylogeny of basal fungi and 14-3-3 proteins from *Homo sapiens* clearly supports Fm201 as a sister clade to Ri14-3-3 (**Figure 1A**), indicative of the conserved evolutionary origin of the *14-3-3* genes in AM fungi, whereas the RiBMH2 protein from *R. irregularis* belongs to the closer relative of the yeast BMH2 (**Figure 1A**). Compared to the 14-3-3 proteins from *H. sapiens*, Fm201 protein still shares a very high homology. This also demonstrates that 14-3-3 proteins are highly conserved in eukaryotes. As a conserved protein, Fm201 protein shares 97% similarities with Ri14-3-3 protein from AM fungi. The amino acid sequence of Fm201 was compared with BMHs from *S. cerevisiae*, 14-3-3s from *R. irregularis* and *H. sapiens* 14-3-3 epsilon and a high homology with over 72% identity at the amino acid level was observed (Supplementary Figure S2).

We further carried out the Homology modeling using *Homo sapiens* 14-3-3 epsilon (80.52% identity in amino acid sequences) as a model. The predicted three-dimensional conformation of Fm201 indicates that Fm201 is a typical 14-3-3 protein with 9 alpha helices and 8 loops, with the highly homologous α3, α5, α7, and α9 putatively forming its amphipathic ligand-binding grooves (**Figure 1C**). Fm201 homologous dimers could form a typical C-shape cup, which provides a basic structure of 14-3-3 dimers for implementing its function (**Figure 1D**).

### 14-3-3 Proteins Restore Metabolic Activity of *S. cerevisiae Δbmhs* Mutant

**FIGURE 2:**
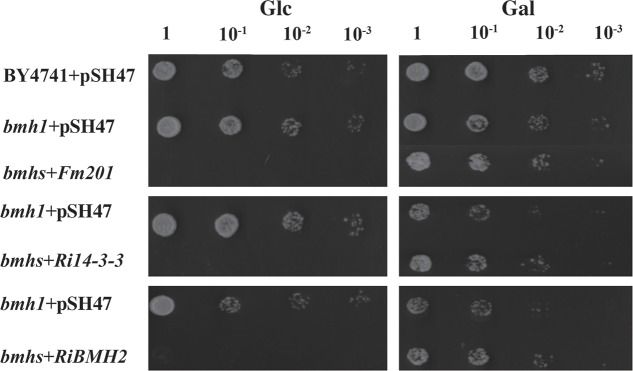
14-3-3 proteins from mycorrhizal fungi restore the growth of *S. cerevisiae Δbmhs* mutant on galactose-contained media. Ten-fold serial dilutions of yeast cells (the wild-type BY4741, Δ*bmh1* mutant or Δ*bmhs* mutant) carrying different constructs (the empty vector pSH47, the full-length cDNA of *Fm201, Ri14-3-3, or RiBMH2*) were grown on SD/-Ura plates with 2% glucose or galactose as carbon source. Expression of *Fm201*, *Ri14-3-3*, or *RiBMH2* gene is controlled by galactose-inducible Gal7 promoter.

To gain further insights into the function of 14-3-3, a *S. cerevisiae* heterologous expression system was exploited. Since Fm201 shares 82.3% identity at the amino acid sequence with both BMH1 and BMH2 in *S. cerevisiae* (see Supplementary Figure S2), the ORF of *Fm201* was cloned into pMR-12 under the control of the *Gal7* promoter and replaced *S. cerevisiae BMH1*. To test if Fm201 can restore the metabolic activity of *S. cerevisiae Δbmhs* mutant, as referred in Materials and Methods (also see Supplementary Table S1). *S. cerevisiae Δbmhs* mutant with pMR-12-*Fm201* cannot grow on YPD with 2% glucose as the sole carbon source. However, cell growth was recovered when using 2% galactose as the sole carbon source (**Figure 2**). Similar results were observed when replacing *Fm201* with *Ri14-3-3* or *RiBMH2* (**Figure 2**). These data confirmed that Fm201 has similar function as BMH1 in *S. cerevisiae*.

### Functional Dissection of CREs on Promoter of *Fm201* (pFm201) in *S. cerevisiae*

14-3-3 proteins have been reported to participate in pseudohyphal growth and resistance in yeast (Roberts et al., 1997; Hurtado and Rachubinski, 2002). However, the roles of these 14-3-3 proteins in mycorrhizal fungi are largely unknown. It has been observed that expression specificity of plant 14-3-3 genes in response to various stresses is largely promoter dependent (Aksamit et al., 2005). To analyze the putative CREs (Cis-Regulatory Elements) located in the promoter region of *Fm201*, a 1.5 kb length promoter sequence upstream of *Fm201* coding region (pFm201) was analyzed via Yeastract database^1^. The CREs present in pFm201 were compared with pBMH1, pBMH2 from *S. cerevisiae* and pRiBMH2 in *R. irregularis* (van Heusden, 2009) (**Table 1**). Many common CREs in corresponding sites shared by pFm201 and pBMHs have been shown to be recognized by many transcriptional factors (Bruckmann et al., 2004; van Heusden, 2009). In the present study, two CREs possiblely recognized by Msn2 and STE12 were chosen for further investigations. Msn2 is an STRE element (AGGGG/CCCCT) binding transcription factor, which is supposed to be related to fungal infection and resistance to abiotic stress in other filamentous fungi (Schmitt and Mcentee, 1996; Seidl et al., 2004; Elfving et al., 2014; Zhang et al., 2014). STE12, so-called GintSTE, is the transcriptional factor that has been reported in mycorrhizal fungi and is believed to be an indispensable component in the early process of mycorrhizal fungi infection (Tollot et al., 2009; Tang et al., 2016). The common and shared CREs present upstream of *Fm201*, *RiBMH2* and *S. cerevisiae BMHs* imply that 14-3-3 proteins in AM fungi may be involved in the regulation of resistance to abiotic stress and hyphal growth in AM fungi as BMHs in *S. cerevisiae*.

**Table 1:** Predicted motifs on the *Fm201* gene promoter compared with that of *RiBMH2*, *ScBMH1* and *ScBMH2^∗^*

Transcription factors	Motif	*ScBMH1*	*ScBMH2*	*Fm201*	*RiBMH2*
Ash1p	YTGAT	-510F	-955F, -881R, -1173R, -1252R	-951F, -1164F, -522R, -664R, -748R, -794R, -1098R, -1164R, -1200R,-1216R, -1238R	-1094F, -1071F, -1034F, -977F, -616F, -436F, -165F, -131F, -157R, -354R, -491R, -789R, -932R, -1178R
Bas1p, Gcn4p	TGACTC	-324R			-403F, -800R
Cbf1p	RTCACRTG	-388R		-1285F	
Fkh1p, Fkh2p	RYMAAYA	-300F, -512R, -520R	-981F, -955R, -965R, -997R	-22F, -277F, -717F, -721F, -1387F, -1499F, -774R, -1552 R	-1491F, -1453F, -1118F, -945F, -599F, -514F, -505F, -496F, -492F, -425F, -369F, -344R, -523R, -1132R, -355FR
Gcn4p	TTGCGCAA	-506FR			
Gcn4p	CACGTG	-389FR		-937FR	
Gcr1p	CWTCC	-292F, -314R, -348R, -378F	-1074F, -924F	-384F,-279R,-1284R, -1482R	-313R
Mot3p	TMGGAA	-67R	-1361F, -1283R		-1320F, -1312F,
Mot3p	AAGAGG	-290R, -316F, -376R	-1024R		
Mot3p	AAGGWT		-1300R	-221F,-856F,-1020F, -1129R	-1326F, -378F
Nrg1p	CCCTC		-921F	-1037R	
**Msn2p^∗∗^**	**CCCCT**	**-212R, -420R, -429R**		**-490F**	**-985F, -1173R**
Pho4p	CACGTK	-389FR			
Rgt1p	CGGANNA	-335R	-1360F, -1073R	-1070F, -786R	-1127F
Rpn4p	GGTGGCAAA	-304F			
Rtg1p, Rtg3p	GTCAC	-388R	-1069R	-1270F,-1285F	-147F, -249R, -185R
Stb5p	CGGNS	-270F, -277F, -282R, -305F, -330F, -382F, -539F	-1078F, -1055F, -1127R	-121F, -345F, -1070F,-87R,-1435R,-1535R	-1189F, -308F, -301F, -289F, -262F
**Ste12p^∗∗^**	**TGAAACA**		**-1222R**	**-1322R,**	**-1159R**
Tec1p	CATTCT		-873F, -1381F	-969F, -329R, -963R	-1080R
Yap1p	TKACAAA	-187f	-715F	-861R	-521R


*^∗^1.5 kb length promoter sequences of RiBMH2 and Fm201 were chosen to analysis the predicted motifs. The promoter sequence of *Ri14-3-3* has not yet been released. ^∗∗^ Words in bold are transcription factor verified in the report.*

**FIGURE 3:**
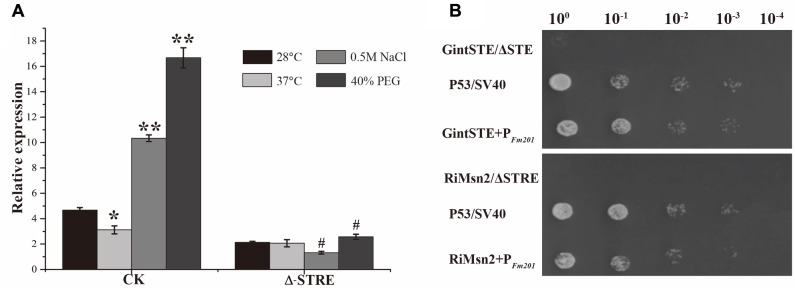
Promoter activity analysis of *Fm201* gene in Yeast. **(A)** Real-time RT-PCR quantification of *Fm201* gene expression in yeast under different abiotic stress treatments. CK indicates the full-length promoter sequence of Fm201 gene. Lines with a significant ratio to the express rate of each group in 28°C. Error bars indicate the means of three biological replicates with SD values. Data shown are averages ± SD; *n* = 3. (#, ^∗^*p* < 0.05, ^∗∗^*p* < 0.01). **(B)** Yeast one-hybrid analysis of the interaction between GintSTE or RiMsn2 with *Fm201* promoter (pFm201). Yeast carrying both pGBKT7-P53 and pGADT7-SV40 was used as the positive control. pHis2- ΔSTRE with pGADT7-Rec2-RiMsn2 and pHis2- ΔSTE with pGADT7-Rec2-GintSTE were used as negative controls. Δ-STRE indicates the deletion of STRE elements located in pFm201 promoter, Δ-STE indicates the deletion of GintSTE binding sites located in pFm201 promoter. 10-fold serial dilutions of yeast cells were spotted on plates containing 2% Glc as carbon source.

Due to the lack of stable genetic transformation approaches in the AM fungi, it is technically challenging to knockout *Fm201* gene to confirm the biological function in the early stages during symbiosis (Sanders, 1999; Maldonado-Mendoza et al., 2001). To probe the possible function of Fm201 protein, we employed the site-specific mutagenesis and yeast one-hybrid system to initially explore the essential region of *Fm201* promoter. Compared to the site-specific mutagenesis of STRE (CCCCT/AGGGG) located in pFm201(pFm201-ΔSTRE), pFm201 is more sensitive to abiotic stresses, osmotic pressure and drought stress (**Figure 3A**). This result indicates that the STRE element of pFm201 could be recognized by Msn2 in *S. cerevisiae*. Although there is no any report, to our knowledge, about the functional properties of RiMsn2 factor mentioned as RiMsn4 in *R. irregularis*, it may play a major role in eukaryotic abiotic stress response and hyphae differentiation (Tisserant et al., 2013; Zhang et al., 2014).

A yeast one-hybrid system was also performed to confirm the interaction between GintSTE and RiMsn2 with CREs on pFm201 (**Figure 3B**). As shown in **Figure 3B**, yeast cells harboring pFm201 with STRE element and RiMsn2 or GintSTE protein grew well, whereas growth of the cells carrying RiMsn2 or GintSTE and *Fm201* promoter without STRE element as negative controls were severly inhibited under the same conditions. These data suggest that GintSTE and RiMsn2 proteins interact with the STRE element of *Fm201* gene, the similar results were also acquired from the promoter of *RiBMH2* (data not shown). It is therefore reasonable to speculate that GintSTE and RiMsn2 proteins positively regulate the expression of *14-3-3* genes in presymbiotic stage through the binding between GintSTE/RiMsn2 and pFm201.

### *14-3-3* Genes Are Highly Induced in Germinating Spores and Early Stages of Symbiosis

**FIGURE 4:**
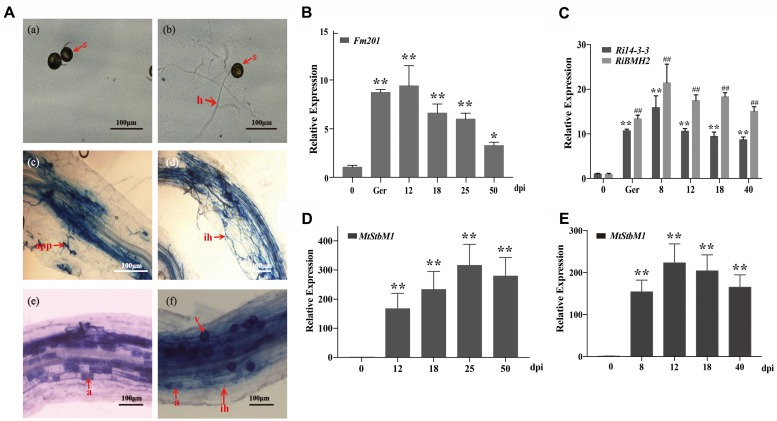
Transcript profiles of 14-3-3 genes in quiescent spores, germinating spores and during different symbiotic stages. **(A)** Optical micrographs of the morphological structures of *F. mosseae* BEG12 during different stages of mycorrhizal symbiosis. Spore (s), hyphae (h), appressorium (app), arbuscule (a), and Vesicle (v) are shown. Scale bars represent 100 μm. **(B)** Expression fold change of *Fm201* from the AM fungus *F. mosseae*. Transcript abundance of *Fm201* was evaluated by real time RT-PCR in different fungal tissues: quiescent spores (0), germinating spores (ger), uninfected *M. truncatula* roots (Uninfected) and mycorrhizal roots (12–50 dpi). *Fm201* gene is expressed as a ratio relative to *FmActin* gene from *F. mosseae.* Lines with a significant ratio to the express rate of *Fm201* in quiescent spores. **(C)** Expression fold change of *Ri14-3-3* and *RiBMH2* from *R. irregularis*. Transcript abundance of *Ri14-3-3* and *RiBMH2* was evaluated by qRT-PCR in different fungal tissues: quiescent spores (0), germinating spores (ger), uninfected *M. truncatula* roots (Uninfected) and mycorrhizal roots (8–40 dpi). *Ri14-3-3* and *RiBMH2* gene is expressed as a ratio relative to *RiActin* gene from *R. irregularis.* Lines with a significant ratio to the express rate of *Ri14-3-3* or *RiBMH2* in quiescent spores. **(D)** The transcript abundance of *MtSbtM1* in *M. truncatula* relative to *MtTEF* in mycorrhizal roots infected by *F. mosseae* (12–50 dpi). **(E)** The transcript abundance of *MtSbtM1* in *M. truncatula* relative to *MtTEF* in mycorrhizal roots infected by *R. irregularis* (8–40 dpi). Error bars indicate the means of three biological replicates with SD values. Data shown are averages ± SD; *n* = 3. (#, ^∗^*p* < 0.05, ##, ^∗∗^*p* < 0.01).

Due to the obligate biotrophic and asexual multinucleate nature of the AM fungi (Sanders, 1999; Maldonado-Mendoza et al., 2001), it is difficult to generate mutants and overexpression strains to analyze the biological functions of Fm201 protein during AM fungal infection. To obtain further insights into the expression profile of *Fm201* gene during the colonization process, we performed a time-course analysis of *Medicago truncatula* roots inoculated with *F. mosseae* in the pot system, then sampled at 12, 18, 25, and 50 days post-inoculation (dpi) and also collected the quiescent spores and germinated spores. Morphological analyses of mycorrhizal roots showed almost majority of the appressoria and intraradical hyphae at 12–18 dpi. More arbuscules were obviously detected starting from 25 dpi, while the abundance of arbuscules decreased at 50 dpi (**Figure 4A**), the mycorrhizal colonization of the root samples was also calculated as described by Trouvelot et al. (2015) (data not shown). The transcript abundance of *Fm201* gene in *F. mosseae* at different stages were also analyzed by qRT-PCR. As shown in **Figure 4B**, the transcript abundance of *Fm201* was obviously higher in germinating spores than in quiescent spores, the transcript abundance of *Fm201* is ∼40% lower in 50 dpi than in 25 dpi when arbuscules decreased. This expression pattern was similar with fungal colonization in early stages of symbiosis, especially during the hyphopodium formation and root penetration (see **Figures 4A,D**). The similar results of *RiBMH2* and *Ri14-3-3* were also obtained from quiescent spores, germinated spores and sampled at 8, 12, 18, 40 days post-inoculation (dpi) by *R. irregularis* (**Figure 4C**). The expression levels of both *RiBMH2* and *Ri14-3-3* obviously increased accompanying the infection process and arbuscules initiation, as demonstrated by the parallel increased transcriptional levels of *MtStbM1* (**Figure 4E**), the host plant subtilase-encoding gene which is considered as a molecular marker of arbuscular mycorrhiza development (Wegel et al., 2007; Takeda et al., 2009).

### Knock-Down of *14-3-3* Impairs the Arbuscule Formation in AM Symbiosis

**FIGURE 5:**
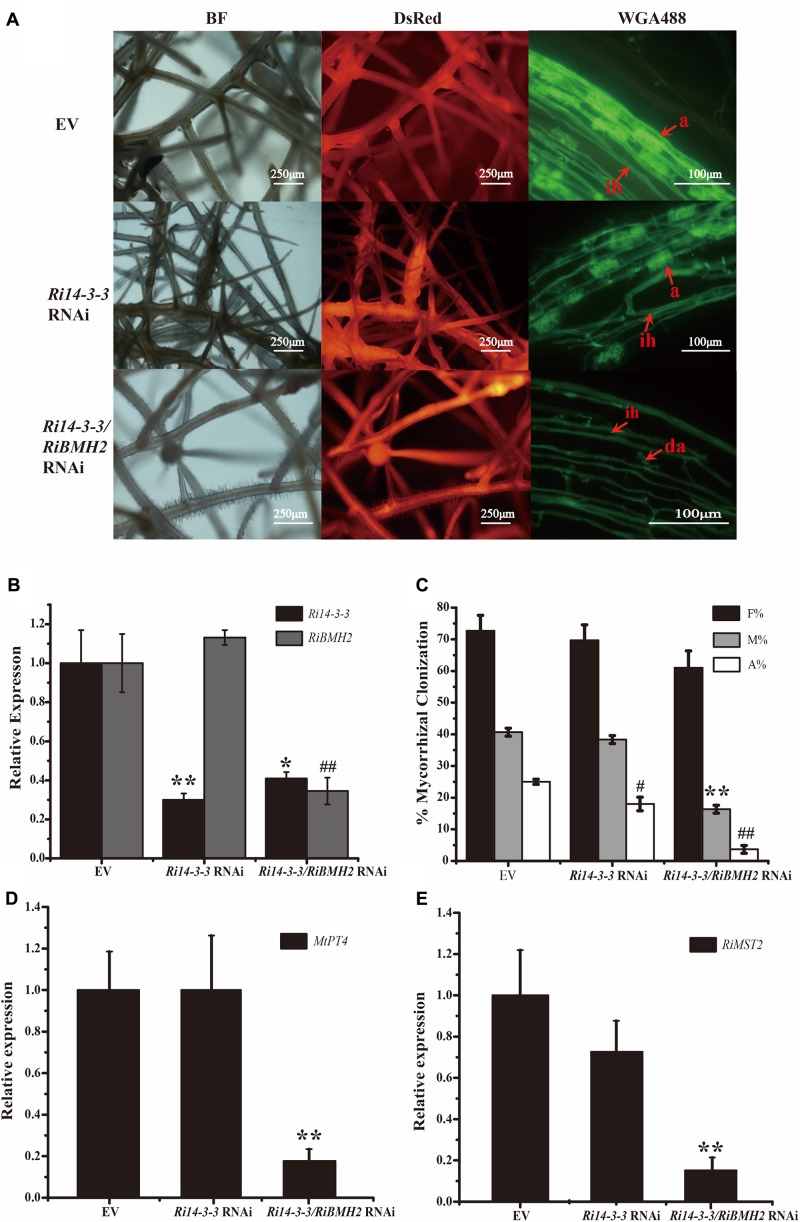
Mycorrhizal symbiotic phenotypes of Host-Induced Gene Silencing of *Ri14-3-3* and *RiBMH2*. **(A)** Hairy root transformation of *M. truncatula* with empty vector (EV), *Ri14-3-3* RNAi vector, or *Ri14-3-3*/*BMH2* RNAi vector. Transgenic hairy roots were infected by AM fungi and the mycorrhizal phenotypes were observed with fluorescence microscope. a, mature arbuscules; ad, arbuscule degradation; ih, internal hyphae. **(B)** Transcript abundance change of *Ri14-3-3* and *RiBMH2* in transgenic hairy roots as measured by qRT-PCR using *RiActin* gene as the reference gene. **(C)** Mycorrhization level was analyzed by WGA 488 staining of hairy roots at 30 dpi with *R. irregularis*. F%, frequency of colonization; M%, intensity of mycorrhiza; A%, arbuscule abundance. **(D)** Expression levels of *MtPT4* in control (EV) and RNAi lines were determined by real-time RT-PCR. The *M. truncatula MtTEF* gene was used as the reference gene. **(E)** Transcript accumulation of *RiMST2* in control (EV) and RNAi mycorrhizal roots measured by real-time RT-PCR. The *R. irregularis RiActin* gene was used as endogenous control. Three technical replicates were analyzed. Asterisks indicate statistically significant differences from respective control lines. Error bars indicate the means of three biological replicates with SD values. Data shown are averages ± SD; *n* = 3. (#, ^∗^*p* < 0.05, ##, ^∗∗^*p* < 0.01).

Because of the obligate property of mycorrhizal fungi, the effect of Δ*Fm201* mutant on the establishment and maintenance of mycorrhizal symbiosis cannot be confirmed *in vivo*. RNAi technique has been successfully utilized to inhibit *Ri14-3-3* encoding a homologous protein of *Fm201* as described above in *R. irregularis* induced by hairy root lines of *M. truncatula* during symbiosis (Nowara et al., 2010; Helber et al., 2011). A 262 bp cDNA sequence from *R. irregularis Ri14-3-3* gene was cloned into pK7GWIWG2 (II) RR according to the approach mentioned in Materials and Methods. The Host-Induced Gene Silencing (HIGS) results of *Ri14-3-3* showed no significant influence on the intraradical structures of *R. irregularis* within the roots, when compared with the control roots (**Figure 5A**).

Since the draft of *R. irregularis* genome was recently released (Tisserant et al., 2013; Lin et al., 2014), we cloned and identified the coding sequence of another 14-3-3 protein subunit termed *RiBMH2* (EXX69786.1). The existence of this novel 14-3-3 protein subunit may explain the nice arbuscule observed in HIGS of *Ri14-3-3*. Thus, the HIGS experiment targeting both *Ri14-3-3* and *RiBMH2* was designed to address this issue. Mycorrhizal phenotype analysis uncovered that the arbuscules are defective. The almost collapsed arbuscules were present in the hairy root of *Ri14-3-3/RiBMH2* RNAi plants, when these two genes were both strongly repressed. Furthermore, the mycorrhizal colonization of each group was also calculated. The data suggests that the abundance of arbuscules in RNAi roots was also significantly lower than that in the control roots (**Figures 5A–C**). Moreover, the expression levels of symbiotic *MtPT4* and *RiMST2*, which are considered as molecular markers of the functioning of arbuscules (Harrison et al., 2002; Helber et al., 2011), are significantly reduced in the *Ri14-3-3/RiBMH2* RNAi roots relative to the control roots (**Figures 5D,E**), indicating that knock-down of both *Ri14-3-3* and *RiBMH2* has a significant effect on the symbiotic phenotype of AM symbiosis. These results also suggest that RiBMH2 may be required for arbuscule formation in AM symbiosis. It also provides a direct evidence that AM fungal 14-3-3 proteins play important roles during AM symbiosis.

### *14-3-3* Genes Are Up-Regulated in Response to Salinity and Drought Stresses During AM Symbiosis

**FIGURE 6:**
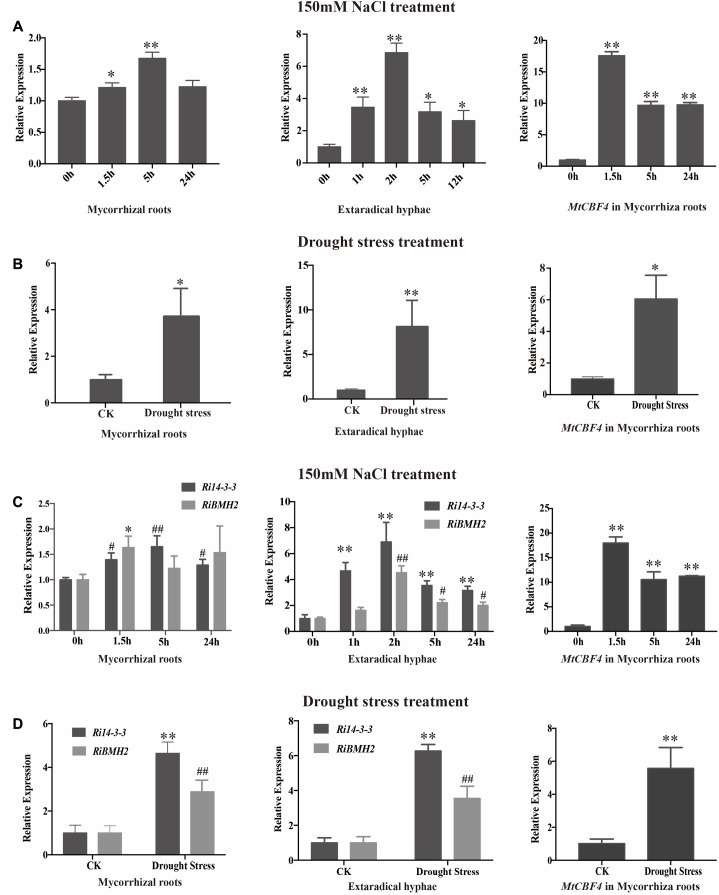
The transcript profile of *14-3-3* in AM symbiosis under drought and osmotic stresses. **(A)** Expression fold change of *Fm201* and *MtCBF4* in mycorrhizal roots, external hyphae or after exposure to osmotic stress treated by 150 mM NaCl for different hours. **(B)** Expression fold change of *Fm201* and *MtCBF4* in mycorrhizal roots, external hyphae under drought stress. **(C)** Expression fold change of *Ri14-3-3, RiBMH2* and *MtCBF4* in mycorrhizal roots, external hyphae after exposure to osmotic stress treated by 150 mM NaCl for different hours. Lines with a significant ratio to the express rate of *Ri14-3-3, RiBMH2 or MtCBF4* in 0 h. **(D)** Expression fold change of *Ri14-3-3, RiBMH2*, *MtCBF4* in mycorrhizal roots, external hyphae. The *FmActin*, *RiActin* or *MtTEF* was used as the reference gene. Three biological replicates were analyzed. Asterisks indicate statistically significant differences from respective control lines. Lines with a significant ratio to the express rate of *Ri14-3-3, RiBMH2 or MtCBF4* in CK. Error bars indicate the means of three biological replicates with SD values. Data shown are averages ± SD; *n* = 3. (#, ^∗^*p* < 0.05, ##, ^∗∗^*p* < 0.01).

To further investigate the potential roles of 14-3-3 proteins in response to salinity and drought stresses, the transcript profiles of *14-3-3s* in mycorrhizal roots and external hyphae were analyzed by qRT-PCR after 150 mM NaCl treatment for various time (**Figures 6A,C**). The transcript abundance of *14-3-3* shows slight but significant increase after 1.5 h and relatively stable within 24 h in mycorrhizal roots. In addition, the transcription profile of *Fm201* in extraradical hyphae treated with NaCl shows more rapid induction than in intraradical mycelia. To determine whether *14-3-3* genes are responsive to drought stress, the transcript abundance of *14-3-3* genes under 1/2 water holding capacity of drought treatment was also compared (**Figures 6B,D**). Unlike salinity stress treatment, the transcript abundance of *14-3-3* genes show a ∼4 fold and ∼7 fold up regulation in mycorrhizal roots and extraradical hyphae, respectively. These findings suggest that *Fm201* may be responsible for the crosstalk between plant and *R. intraradices* under salinity and/or drought stresses.

## Discussion

In terrestrial ecosystems, AM symbiosis is considered to be the most widespread ecologically and agriculturally mutualistic beneficial association among plant symbioses. Despite their great importance in both ecology and agriculture, advance in understanding the molecular basis of AM symbiosis from the fungal aspect is slow until the release of the transcriptomic data of several AM fungal species (Tisserant et al., 2012; Salvioli et al., 2016; Tang et al., 2016) and genomic data of *R. irregularis* (Tisserant et al., 2013; Lin et al., 2014), biological functions of only a few genes have been characterized during interaction with plants. In such a context, we focus on the characterization of the *14-3-3* genes from AM fungi based on its higher expression during the colonization process.

### AM Fungal 14-3-3 Proteins Are a Conserved Feature of Glomeromycota

According to bioinformatics analyses, 14-3-3s from AM fungi are typical 14-3-3 proteins with higher similarity to the known 14-3-3 sequences of yeast and human. Additionally, Fm201 protein is conserved across eukaryotes based on the phylogenetic relationships among AM fungi and other basal eukaryotic species as well as the conserved 3D homology structures between *F. mosseae* and human (Yang et al., 2006). Therefore, it is of interest to find that two similar sequences were found in the recently released genome and transcriptome of another AM fungus, *R. irregularis* (Tisserant et al., 2012, 2013). Since the sequences of the two additional genes, the so called *Ri14-3-3* and *RiBMH2*, are complete with the full-length of CDSs, and the percentage of identity is relatively high (∼97%), the three AM fungal proteins share the same nine α-helix domain topologies. Among them, *Ri14-3-3* gene from *R. irregularis* has been firstly reported by Porcel et al. (2006). Moreover, RNA-seq data presented a significant induction *in planta* phase compared to spores (Tisserant et al., 2012). Only the investigation within genomic and transcriptomic data in AM fungi will clarify whether *Fm201*-related sequences are a general feature among fungi. Consistent with the previous *in silico* analyses, these three Fm201, Ri14-3-3 and RiBMH2 are able to complement the yeast *BMH1* and *BMH2* double mutants. This finding is in agreement with those data reported in the earlier studies (van Heusden et al., 1995, 1996), indicating that these genes identified above encode the functional 14-3-3-like proteins in AM fungi. Further studies need to be carried out to confirm whether these 14-3-3-like proteins identified are a conserved feature of Glomeromycota and whether they may have an essential role in the intraradical phase during interaction with the host plants.

The transcription of *14-3-3* genes show a clear increase in the germinating spores as well as the intraradical phase in both *R. irregularis* and *F. mosseae*. The data stemming from the time-course experiment presented that the relatively higher transcription levels were achieved in the phases of root penetration and arbuscules formation, while the expression levels of *Fm201* and *Ri14-3-3* are obviously reduced compared with *RiBMH2* in the degenerating mycorrhizal roots. In addition, we also correlated the *Fm201* mRNA abundance with the morphological structures of *F. mosseae* inside the roots (at 12–50 dpi). The results of *Fm201* transcription patterns also suggest that it may play an important role in the germination and hyphopodium formation of *F. mosseae*, which was also proposed by Breuninger and Requena (2004) through SSH of AM symbiosis at the early stage. In addition, transcript levels of *Fm201* remain higher during the symbiotic stage (see **Figure 4B**), suggesting that this 14-3-3 protein may also play important roles during AM symbiosis, especially the formation of arbuscule besides the root penetration stage. It is thus speculated that the expression of *14-3-3s* are, to some extent, related to root penetration and arbuscules formation. This hypothesis is supported by the evidence that *14-3-3* transcripts were present in both the laser micro-dissected arbuscule-containing cells and the IRM including intercellular hyphae (Tisserant et al., 2012). Overall these data implicate a relationship between AM fungal 14-3-3 related genes and intraradical hyphal growth and arbuscule differentiation.

### Two AM Fungal 14-3-3 Protein Subunits Have the Impacts on the Success of Arbuscular Mycorrhizal Colonization and Arbuscule Formation

The potential involvement of AM fungal 14-3-3 genes *Ri14-3-3* and *RiBMH2* in the *in planta* phase of the colonization process was also supported by the HIGS of *Ri14-3-3* and/or *RiBMH2* during the *M. truncatula*–*R. irregularis* mycorrhizal symbiosis. Lacking the stable genetic transformation protocols for AM fungi, HIGS was confined to AM fungi (Helber et al., 2011; Xie et al., 2016).

The data of the knock-down of both *Ri14-3-3* and *RiBMH2* genes by HIGS resulting in the impaired arbuscule formation of *R. irregularis* suggest the significance of these AM fungal 14-3-3 proteins for AM symbiosis. Connecting with the transcripts of *RiBMH2* during *M. truncatula–R. irregularis* mycorrhizal symbiosis, *RiBMH2* may be required for the development of AM symbioses and the arbuscule differentiation within roots. However, the *Ri14-3-3* RNAi roots colonized by *R. irregularis* exhibited a considerable arbuscule abundance as compared with control mycorrhizal roots. These findings suggest that the AM functionality or arbuscule formation is redundantly regulated by the two 14-3-3-like genes in *R. irregularis*. Nevertheless, we here propose that *RiBMH2* is essential for arbuscule formation, whereas *Ri14-3-3* could be involved in the colonization process but not AM functionality. This hypothesis is supported by the evidence that the transcripts of *MtPT4* and *RiMST2*, two symbiotic genes responsible for arbuscule functionality, were strongly reduced in *Ri14-3-3*/*RiBMH2* RNAi roots, while they were not repressed in *Ri14-3-3* RNAi roots. Although *Ri14-3-3* homologous gene *RiBMH2* is identified in the *R. irregularis* draft genome (Tisserant et al., 2013; Lin et al., 2014) and *RiBMH2* was not down-regulated in *Ri14-3-3* RNAi roots (see **Figure 5B**), the normal AM fungal structures observed in this HIGS system indicate a novel but unknown role for *Ri14-3-3* in the establishment of AM symbiosis. Based on the above findings and the previous study (Liu et al., 2015), we hypothesize that RiBMH2-mediated signal could be an important signal in the control of arbuscules formation and *R. irregularis* hyphal growth within roots. This unknown signal relayed by RiBMH2 serves as the essential signal to ensure the metabolic activity of *R. irregularis* in the hyphal growth and/or arbuscule differentiation during symbiosis. In the absence of this RiBMH2-mediated signal, the arbuscules are impaired, and growth of the fungus is prevented. The *R. irregularis* itself needs to activate 14-3-3 protein RiBMH2 in response to the environmental clues to meet demands during fungal growth and division. In addition, our functional analysis in yeast cells suggested that *Ri14-3-3* and *RiBMH2* encode functional signal proteins involved in growth induction (see **Figures 2B,C**), indicating that these two proteins may play potential roles in signal transduction during the colonization process and arbuscule formation, respectively. Thus, we can speculate the involvement of Ri14-3-3 in fine-tuning fungal growth in the intraradical phase responding to the external stimuli, moreover, RiBMH2 may be indispensable for arbuscules differentiation. This complex mechanism by which arbuscular mycorrhizas are formed in roots requires the elaborate control of the two AM fungal 14-3-3 proteins in the intraradical phase during cross-talk with host plant.

Remarkably, these results from the HIGS experiments revealed that one 14-3-3 protein subunit can adjust its own expression quantity to offset the adverse influence caused by the lack of another 14-3-3 protein subunit. This conclusion is consistent with the previous results derived from yeast system (van Heusden et al., 1995). Based on this point, it is reasonable to hypothesize that the AM fungal 14-3-3 proteins are indispensable for the symbiosis functioning.

### Involvement of AM Fungal 14-3-3 Proteins in Msn2/STRE Element-Mediated Signaling Pathway

The knockdown of both *Ri14-3-3* and *RiBMH2* by HIGS exhibits somewhat distinct phenotypes, i.e., fewer arbuscule abundance and impaired arbuscules (see **Figures 5A,C**), repression of the endosymbiosis functioning with regard to transcription of the symbiotic *MtPT4* and *MST2* genes (see **Figures 5D,E**). We hypothesize that there exists a positive feedback mechanism in the potential signaling pathway in *R. irregularis*. It is also proposed that the CREs upstream of a gene always show close relationship with its function, especially for the regulatory proteins (Carey et al., 2012; Petrov et al., 2012), although most CREs are composed of short sequences which may be very abundant in eukaryotic genomes (van Heusden, 2009). As expected, we observed some conserved motifs including STRE elements in the promoters of two AM fungal 14-3-3 genes *Fm201* and *RiBMH2* (see **Table 1**), as predicted by YEASTRACT database, in comparison with the promoters of yeast *BMH1* and *BMH2*. Interestingly, the deletion of STRE (CCCCT/AGGGG) element in the promoter of Fm201 showed significantly reduced levels of the reporter gene mRNAs when expressed in yeast cells (see **Figure 3A**). These hypotheses mentioned above are also supported by the fact that the orthologous *Fm201* gene promoter with STRE element (pFm201) directly interacts with transcription factor Msn2 in yeast cells (see **Figure 3B**). Furthermore, this recognition between RiMsn2 and pFm201 may contribute to the induction of *Fm201* in extraradical hyphae in response to the salinity (150 mM NaCl treatment) stress (see **Figure 6A**). Therefore, based on the site-specific mutagenesis and the yeast one-hybrid analyses, the transcription of AM fungal 14-3-3-like genes during AM symbiosis is Msn2/STRE-element dependent. The zinc finger DNA-binding proteins Msn2 and Msn4 serve as the key factors that controlling fungal growth and stress responses in different fungal species (Martinez-Pastor et al., 1996; Schmitt and Mcentee, 1996; Liu et al., 2013; Zhang et al., 2014). In addition, the Msn2-controlled and STRE-driven gene *Fm201* and *RiBMH2* from *F. mosseae* and *R. irregularis*, respectively, are positively regulated in response to drought stress during AM symbiosis (see **Figures 6B,D**), reinforcing that AM fungal 14-3-3 genes participate in the Msn2/STRE element-mediated signaling pathway in AM fungal symbiont during AM symbiosis.

**FIGURE 7:**
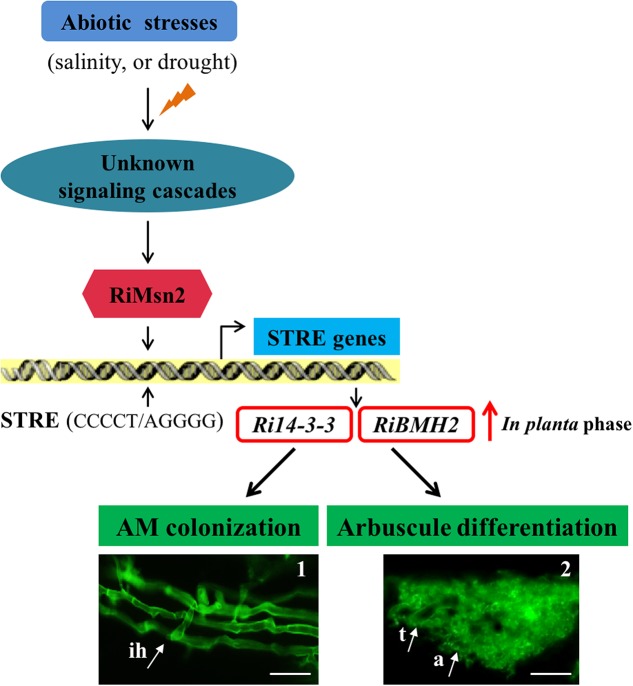
Proposed Working Model of two 14-3-3 proteins in *R. irregularis*. Schematic representation of the abiotic stresses-induced signaling cascades in AM fungi and the involvement of *Ri14-3-3* and *RiBMH2* genes in the RiMsn2/STRE-mediated signaling pathway during AM symbiosis. In AM fungi, the unknown signaling cascades are triggered by the external stress stimuli such as salinity or drought; then the core component RiMsn2 factor is activated by the upstream of potential cascades. The functional RiMsn2 is translocated to the nuclear to recognize the STRE (Stress response element) (CCCCT/AGGGG) on the AM fungal genome. Meanwhile, in the *in planta* phase, transcription of the STRE genes, including Ri14-3-3 and RiBMH2, are clearly induced after this interaction between RiMsn2 and STRE. Thus, the expression of Ri14-3-3 and RiBMH2 proteins are involved in or essential for AM colonization and arbuscule differentiation within roots. The black arrows indicate the positive interactions, while the red arrow suggests the induction of *Ri14-3-3* and *RiBMH2* genes in the *in planta* phase. The images 1 and 2 represent the intraradical hyphae and arbuscule from WGA488 staining. ih, intraradical hyphae; t, trunk; a, arbuscule. Scale bars represent 25 μm.

Overall these data presented in this study provided new insights into the signaling function of the 14-3-3 proteins in AM fungal cells during crosstalk with host plants. Based on the aforementioned data, we also propose the hypothesis that abiotic stresses such as salinity and drought affect a Msn2/STRE-mediated signaling pathway governing the expression of AM fungal 14-3-3 proteins that promoted fungal colonization and arbuscule formation within roots (see **Figure 7**). In the first version of the scheme for abiotic stresses induced signaling, it has been proposed that 14-3-3 proteins preferentially expressed in the intraradical phase are involved in AM fungal colonization process and arbuscule functionality by the regulation of Msn2/STRE-mediated signaling pathway that may control the fungal growth and arbuscule lifespan during AM symbiosis.

Further studies, such as characterizing the precise roles of the novel *RiMsn2* gene identified in this work, validating the protein-protein interactions in the Msn2-mediated signaling pathway and the biochemical functions of core components of this pathway, and determining the direct evidence of Msn2-dependent mechanisms in *R. irregularis*, are needed to define the underlying stress response mechanisms in AM symbionts. Furthermore, the RNA-seq data and gene expression analyses show that both *R. irregularis* and *Gigaspora margarita* contain multiple distinct MAPK (Mitogen-activated protein kinase)-related proteins (Tisserant et al., 2012; Salvioli et al., 2016; Xie et al., 2016), indicative of the presence of MAPK signaling cascade in AM fungi to respond to external stresses stimuli and adapt to environmental fluctuation. Thus, a major goal in this field will be to uncover a master MAPK protein regulating the AM fungal growth and differentiation during symbiosis under various abiotic stresses.

In summary, we showed that *Fm201*, *Ri14-3-3* and *RiBMH2*, three genes from two different AM fungi, are preferentially expressed in the intraradical phase and may have impacts on the success of AM colonization and arbuscule formation. Our data also presented that Msn2 protein governs the *Fm201* gene transcription, indicating that AM fungal 14-3-3 gene identified is involved in Msn2 factor/STRE element-mediated signaling pathway. Importantly, host-induced gene silencing of both *Ri14-3-3* and *RiBMH2* impairs the arbuscule differentiation within roots, indicating that the two AM fungal 14-3-3 protein subunits are required for arbuscule formation. Additionally, these AM fungal 14-3-3 genes are up-regulated in response to salinity and drought stresses during AM symbiosis. Based on these new findings, we propose that the AM fungal *14-3-3* genes are essential for the interaction between AM fungi and host plants, and are potentially involved in enhancing plant salinity and drought tolerance by Msn2/STRE element-controlled signaling.

## Materials and Methods

### Biological Materials and Growth Conditions

A-grade spores of *R. irregularis* DAOM197198 were purchased from Agronutrition (Carbonne, France). Spores of *F. mosseae* BEG12 were kindly provided by the International Bank of Glomeromycota (IBG, Dijon, France) and collected from *Medicago truncatula* pot cultures by wet sieving to isolate genomic DNA and total RNA. Spores surface sterilized by 2% chloramine T, and then immersed in a solution containing 0.02% streptomycin and 0.02% gentamycin for 10 min (Besserer et al., 2008). Germinated spores of *F. mosseae* were selected from acetone solution containing 10^-8^mol/L GR24 (10^-9^mol/L GR24 for *R. irregularis* in two days) in 25°C dark incubator. Quiescent spores, germination spores and mycorrhizal roots (at 10, 18 25, 50 dpi for *F. mosseae*; 8, 12, 18, 40 dpi fo*r R. irregularis*) were harvested. After washing in sterile water, all materials described above were immediately frozen in liquid nitrogen and stored at -80°C before nucleic acid extraction.

The water-holding capacity of the soil was computated before planting. The soil was weighed before plant, watered uniformly until flowed from the bottom. Keep the pot suspended in midair for 1 day before weighing. The increase weight of soil after waterd is water-holding capacity. *M. truncatula* mycorrhizal roots inoculated with AM fungi were treated with NaCl (0.5 M) to 150 mM in final concentration (Calculate according to 70% of water holding capacity in soil) (Giovannetti et al., 2001; Li et al., 2011). Drought treatment was measured by 1/2 water-holding capacity treatment which was proved drought stress treatment to *M. truncatula* in previous experiments. Mycorrhizal roots and extraradical hyphae from sandwich system were harvested at 1.5, 5, 24 h after treatments (Abdel Latef and Chaoxing, 2011; Estrada et al., 2012) to monitor the transcript profiles of *14-3-3* genes under different abiotic stresses by qRT-PCR analysis.

*Fm201* promoter-YGFP chimeric gene in *S. cerevisiae* BY4741 was treated with NaCl (500 mM), CuSO_4_ (50 mM), CdCl_2_ (0.2 mM), PEG4000 (25%), and 37°C abiotic stresses in YPD medium and then harvested after 1h treatments (Vido et al., 2001).

### DNA and RNA Extraction, RT-PCR and Real Time RT-PCR

The total DNA was isolated from AM fungal sporescarps as described by Zézé et al. (1994). Total RNA of different AM fungal tissues was extracted with TRIzol reagent (Invitrogen) according to the protocol. Surface-sterilized spores were placed into 1.5 ml RNase free microtube and then frozen in liquid nitrogen, 0.3ml TRIzol solution was immediately added to the microtube. Electric mill (TIANGEN OSE-Y20, Beijing, China) and Phase Lock Gel (TIANGEN, Beijing, China) were used to make sure the quality of RNA. Total RNA yields and concentrations were measured by the Thermo NanoDrop 2000 spectrophotometer (Thermo). To remove residual genomic DNA, each total RNA sample was treated with RNase-free DNaseI (Thermo) according to the manufacturer’s instructions. The first cDNA strand was synthesized as described in RevertAid First Strand cDNA Synthesis Kit (Thermo).

Transcript profiles of AM fungal genes *Fm201*, *Ri14-3-3*, and *RiBMH2* as well as host plant genes *MtSbtM1* and *MtCBF4* in different symbiotic stages and under abiotic stresses were studied by qRT-PCR using ViiA 7 system (Life Technologies, United States), three biological replications were performed. The expression levels were normalized to transcripts of the β-*actin* gene of *F. mosseae* or *R. irregularis* and to transcripts of the *MtTEF* gene of *M. truncatula* (Hohnjec et al., 2005). Before real time RT-PCR, gene-specific primers for all target genes were validated on genomic DNA and cDNA. Total RNA was isolated from AM roots comprised plant and fungal materials. The specificity of the primer pairs were also confirmed via PCR method on *M. truncatula* total DNA. No amplification signals were present on plant DNA. The primers sequences for all genes studied in this work are provided in Supplementary Table S2. qRT-PCR was performed using SYBR Green Real-time PCR Master Mix (TOYOBO, Japan) according to the manufacturer’s instructions. Each 10 μl reaction contained 1 μl of the synthesized cDNA (cDNA pool was diluted to 200 μl), 5 μl SYBR Green Real-time PCR Master Mix, 0.5 μl each primer(10 μM), 3 μl ddH_2_O. PCR program consisted of a 30 s incubation at 95°C to active the hot-start recombinant Taq DNA polymerase, followed by 40 cycles of 10 s at 95°C, 15 s at 57°C, and 20 s at 72°C. The relative levels of transcripts were calculated by using the 2^-ΔΔ*ct*^ method (Livak and Schmittgen, 2001).

### Cloning of *Fm201* Gene From *F. mosseae*

The *Fm201* EST sequence was obtained from NCBI (Accession number: CF803281), (Breuninger and Requena, 2004). Reverse PCR was utilized to get the 5′ flanking sequence of *Fm201* gene. The gene-specific primers 201F and 201R were designed to amplify the partial DNA fragment of *Fm201* according to the available sequence of *Fm201* EST. Genomic DNA of *F. mosseae* was digested by FastDigest restriction enzyme *Xho*I (Thermo). DNA fragments were self-ligated by T_4_ DNA ligase, and the reaction was carried out in a final volume of 20 μl containing 0.5 μl digested DNA fragments, 2 μl 10× buffer, 0.5 μl T_4_ DNA ligase (Thermo), 17 μl ddH_2_O, incubated for 12 h at 10°C. Nest-PCR was performed in this experiment, 0.5 μl ligated production was used as PCR template, the specific primers used in the first PCR reaction were 201RF1 and 201RR1, products from first PCR reaction were diluted to 1/1000 as the template for the second PCR reaction, and specific primer 201RF2 and 201RR2 were used.

RACE as a classic method to rapidly obtain the 5′ and 3′ ends of the *Fm201* gene (Scotto-Lavino et al., 2006a,b). 3′RACE was carried out on the total RNA from *F. mosseae* sporecarps by using primer Q_T_. Two pairs of primers RACE201F/Q_O_ and RACE201F2/Q_I_ were used for the subsequent nest PCR reactions, respectively. Due to the high A/T containing feature of AM fungi genomic DNA, the dGTP and Q_C_ replaced the dATP and Q_T_ used in classic 5′RACE, responsively. The first cDNA strand was obtained from *F. mosseae* sporocarps by using specific primer RACE201R1. Primers Q_C_, Q_o_ and RACE201R2 were used as the first PCR cycle primers, while primers Q_I_ and RACE201R3 were used as the second PCR cycle. Transfast *pfu* DNA polymerase (Transgen, Beijing, China) was used in the PCR reactions mentioned above, PCR products were cloned into pEASY-Blunt vector (Transgen, Beijing, China) and sequenced.

### Plasmids Construction

Plasmid pMR12 was generated from pMRI-11(Xie et al., 2014), the promoter of Gal7 was amplified from the genome DNA of *S. cerevisiae* BY4741 by PCR using the specific primers P_Gal7_F/P_Gal7_R. P_Gal7_ and pMR-11 were digested with both *Sac*I and *Spe*I, respectively, then the digests were cloned into target vector pMR-11. To address the regulation of CREs located in the promoter of *Fm201*, the expression profiles of YGFP reporter were conducted in *S. cerevisiae* BY4741. Monoclonal vector pUG35 carrying a YGFP reporter is used in yeast heterologous systems (Cormack et al., 1997). Two restriction sites were *Sac*I and *Xba*I, which were added to the start codon upstream sequence of *Fm201* (pFm201) by using primers 201PFn (*n* = 1,2,3,4) and 201PR1, then cloned into pUG35 to replace P_MET-25_ to produce a series of 5′ truncated promoters-reporter vectors. pFm201 targeted deletion of *cis*-element was conducted by using SOE-PCR method (Ho et al., 1989). The specific primers used are provided in Supplementary Table S2). The site-directed mutation promoter sequences were also cloned into pUG35 as the promoter truncated verification vectors. qRT-PCR was used for monitoring transcriptional efficiency of *Fm201*, the *Ura* gene of pUG35 was chosen as the internal standard, primers UraF, UraR, YGFPF, YGFPR were used in this experiment (Peter et al., 2006).

### Yeast One-Hybrid Screening

Yeast one hybrid experiment was carried out using the Matchmaker One-hybrid System (Clontech), the ORF of *GintSTE* was cloned from cDNA of *Rhizophagus irregularis* by using primers RiSTE12F and RiSTE12R, and cloned into pGADT7-rec2. Two same 272 bp length promoter fragments contained STE12 targeted *cis*-element STRE were cloned into pHIS2 in tandem, the same fragment only lacking the *cis*-element STRE was also inserted into the pHIS2 as a negative control. Yeast one-hybrid experiment of Msn2 was carried out in the same way and the primers RiMsn2F and RiMsn2R were used as mentioned in Supplementary Table S2.

Plasmids for yeast one-hybrid were co-transformed into yeast Y187 strain. Y187 cells carrying the target plasmids were cultivated in SD medium lacking leucine and tryptophan, and were also gradiently inoculated at 1.0 OD_600_ on SD medium lacking leucine, histidine and tryptophan and supplemented with 30 mM 3-AT, which is a competitive inhibitor of the His3 protein.

### HIGS of *R. irregularis* 14-3-3 Genes in Hairy Root Lines of *M. truncatula*

The RNAi-target sequences of *Ri14-3-3* and *RiBMH2* were amplified by the specific primers Ri14-3-3F/Ri14-3-3R and RiBMH2ATG/RiBMH2F. The PCR products were cloned to the linearized pDONR221 used CloneExpressII (Vazyme, Nanjing, China), then the LR reaction was done to recombine the target sequences into the pK7GWIWG2(II)RR according to the instructions in Gateway protocol.

*Agrobacterium rhizogenes* Msu440-mediated root transformation was performed following the method as described in *Medicago Truncatula* Handbook (International Committee, 2006). *In vitro* hairy roots were cultured on EM plates containing Benzyl penicillin (200 mg/L) for three times. The root tip (2∼3 cm in length) was used for each subculture. The root was re-cultured in the M medium without antibiotics for half a month. The hairy root lines without bacteria were re-cultured in new M medium for mycorrhization. Mycorrhizal hairy root of *P. crispum* without DsRed tag was cut into small pieces (∼3 mm) and placed around the hairy root of *M. truncatula* harboring DsRed marker as described in Supplementary Figure S3. The mycorrhizal roots with red fluorescence were harvested in one month until the external hyphae of *R. irregularis* beyond the hairy root surface of *M. truncatula*.

### Quantification of Arbuscular Mycorrhizal Colonization

Mycorrhizal roots collected from pot cultures were stained with 0.1% Typan blue, while the mycorrhizal hairy roots expressing red fluorescence grown on plates were stained with WGA488, and the estimation of AM colonization was performed as described by Trouvelot et al. (2015) using MYCOCALC program^2^.

### *In Silico* Analysis of Fm201 Protein

The deduced amino acid sequence of Fm201 was analyzed with the computer program DNAstar. Multiple sequence alignments were performed by DNAMAN8. The unrooted phylogenetic tree constructed by neighbor-joining algorithm was carried out using MEGA6. The computation of physical and chemical parameters was conducted by using ProtParam tool^3^. Homology modeling of the three-dimensional structure of Fm201 protein was done with the program Swiss Model^4^ using *Homo sapiens* 14-3-3ε protein (2br9A) as the template (**Figure 1B**; Yang et al., 2006). The *cis*-elements of the promoters *Fm201* and *RiBMH2* were analyzed on YEASTRACT^5^ using *S. cerevisiae* S288c as the reference.

Yeast mutant strains used in this article are constructed with the methods mentioned by van Heusden (van Heusden et al., 1995, 1996). The detail information for each strain is available at Supplementary Table S1. In the construction of *bhms-Fm201*(MATa; his3Δ1; leu2Δ0; met15Δ0; ura3Δ0; BMH1::KanMX (Gal7[*Fm201*]); BMH2::ura3), fragments of pMRI-12 which contain a KanMX and a Gal7 promoter and pSH47 which contain a Ura3 marker were used to replace BY4741, BMH1 and BMH2, respectively, by primers PMRI-12F1, PMRI-12R1 and PSH47F, PSH47R. The *bmhs*-Ri14-3-3 and *bmhs*-RiBMH2 were also built in the same way.

### Statistical Analyses

Statistical analyses were performed through one-way ANOVA. Following ANOVA, Tukey’s test was performed to make comparisons between treatments, using a probability level of *p* < 0.05(^∗^, #), 0.05 ≤*p* < 0.01 (^∗∗^, ##). All statistical analyses were performed using SPSS statistical package (version 23.0, SPSS Inc., United States).

## Accession Numbers

The sequence data can be found in the GenBank data libraries under accession numbers. Nucleic acid sequence: *MtCBF4* (HQ110079.1), *MtStbM1* (XM_003611148.1), *MtPT4* (AY116211.1), *Fmactin* (KM360085.1), *Fm201* (KM258580.1), *Riactin* (EXX64987.1), *RiBMH2* (*JEMT01016782.1*), *Ri14-3-3* (AM049264.1), *RiMST2* (HM143864.1), Ri14-3-3 (CAJ16742.1). Amino acid sequence: Fm201(KM258580), RiBMH2 (EXX69786.1), *R. oryzae* 14-3-3 (EIE87660.1), *M. medusa* 14-3-3 (ABS86241.1), *M. circinelloides* 14-3-3 (EPB82885.1), *A. oryzae* 14-3-3 (XP_001819291.2), *A. niger* 14-3-3 (XP_001399080.1), *S. borealis* 14-3-3 (ESZ95350.1), *M. oryzae* 14-3-3 (XP_003710925.1), *A. nidulans* (CBF81292.1), *A. terreus* 14-3-3 (XP_001212078.1), *P. strigosozonata* 14-3-3 (XP_007382290.1), *S. musiva* 14-3-3 (EMF09853.1), *B. bassiana* 14-3-3 (XP_008601347.1), *R. solani* 14-3-3 (CCO32840.1), *S. cerevisiae* BMH1 (CAA46959.1), *S. tuberosum* 14-3-3 (XP_004250139.1), *O. sativa* 14-3-3 (NP_001047234.1), *S. cerevisiae* BMH2 (CAA59275.1), *R. norvegicus* 14-3-3ε (NP_113791.1), *H. sapiens* 14-3-3ε (NP_006752.1).

## Author Contributions

BZ and XXie conceived this research. ZS and JS prepared the biological material for gene expression analysis. ZS and XXin performed the data analysis. ZS and XXie wrote the manuscript. BZ and XXie revised the manuscript.

## Conflict of Interest Statement

The authors declare that the research was conducted in the absence of any commercial or financial relationships that could be construed as a potential conflict of interest.

## Footnotes

1 http://www.yeastract.com/

2 http://www2.dijon.inra.fr/mychintec/Mycocalc-prg/download.html

3 http://www.expasy.ch/tools/protparam.html

4 http://swissmodel.expasy.org/

5 http://www.yeastract.com/
