# Supplementary material for: Arbuscular Mycorrhizal Fungal 14-3-3 Proteins Are Involved in Arbuscule Formation and Responses to Abiotic Stresses During AM Symbiosis
Source: Front Microbiol. 2018 Mar 5;9:91. doi: 10.3389/fmicb.2018.00091 (PMC5844941; doi:10.3389/fmicb.2018.00091)
Supplement: Supplementary file 1 [file Image_1.pdf]

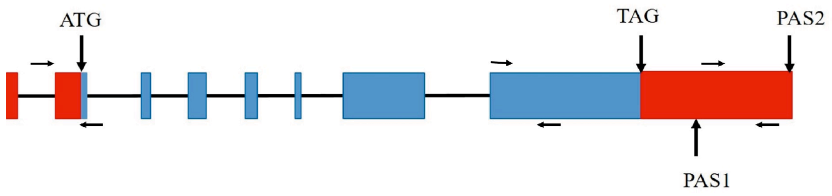

Figure S1. Gene structure of *Fm201*

Fm201 gene contains seven exons and seven introns. Blue boxes stand for exons, red boxes stand for the UTRs, lines between the boxes stand for introns; ATG and TAG indicate start codon and stop codon, respectively. PAS1 and PAS2 stand for the two polyadenylation signals, indicative of two distinct 3' UTRs in *Fm201* gene, while the arrows indicate the specific primers for isolation of the sequence of *Fm201* UTRs.

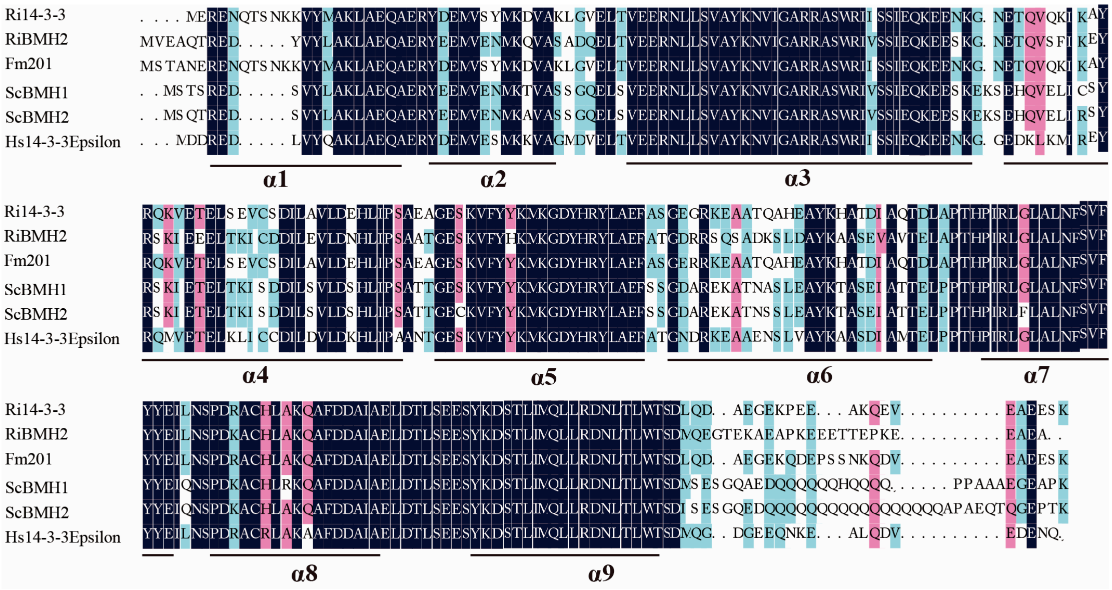

Figure S2. Multiple amino acid sequence alignment among Fm201, Ri14-3-3, RiBMH2, ScBMH1, ScBMH2 and Hs14-3-3ε.

Amino acid sequences with highlighted lines represent 9 predicted α-helixes.

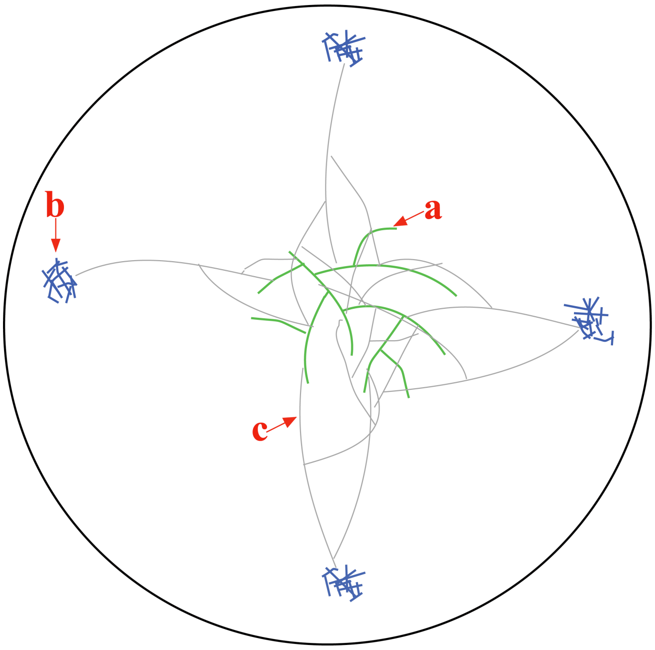

Figure S3. Schematic diagram of HIGS experiment design in petri dishes system.

Culture a represents hairy roots of *M. truncatula* harboring the RNAi vector grown on MS medium. Culture b indicates the infected hairy roots of *Petroselinum crispum* with *Rhizophagus irregularis* as the inocula. Culture c showes the growing extraradical hyphae from mycorrhizal roots of *Petroselinum crispum* toward the roots of *M. truncatula*. 30 days after inoculation, the mycorrhizal roots of *M. truncatula* with Ri14-3-3 RNAi vector were collected for subsequent colonization and gene expression analyses.

Table S1. Yeast Strains used in this study

Strain	Genotype
BY4741	MATa.his3Δ1.leu2Δ0.met15Δ0.ura3Δ0
BY4741-pMRI-12	MATa.his3Δ1.leu2Δ0.met15Δ0.ura3Δ0.KanMX
Bmh1-Fm201	MATa.his3Δ1.leu2Δ0.met15Δ0.ura3Δ0.BMH1::KanMX(Gal7[*Fm201*])
bmh1-Fm201-pSH47	MATa.his3Δ1.leu2Δ0.met15Δ0.ura3Δ0.BMH1::KanMX(Gal7[*Fm201*]).ura3
bmhs-Fm201	MATa.his3Δ1.leu2Δ0.met15Δ0.ura3Δ0.BMH1::KanMX(Gal7[*Fm201*]).BMH2::ura3
bmh1-Ri14-3-3	MATa.his3Δ1.leu2Δ0.met15Δ0.ura3Δ0.BMH1::KanMX(Gal7[*Ri14-3-3*])
bmh1-Ri14-3-3-pSH47	MATa.his3Δ1.leu2Δ0.met15Δ0.ura3Δ0.BMH1::KanMX(Gal7[*Ri14-3-3*]).ura3
bmhs-Ri14-3-3	MATa.his3Δ1.leu2Δ0.met15Δ0.ura3Δ0.BMH1::KanMX(Gal7[*Ri14-3-3*]).BMH2::ura3
Bmh1-RiBMH2	MATa.his3Δ1.leu2Δ0.met15Δ0.ura3Δ0.BMH1::KanMX(Gal7[*RiBMH2*])
bmh1-RiBMH2-pSH47	MATa.his3Δ1.leu2Δ0.met15Δ0.ura3Δ0.BMH1::KanMX(Gal7[*RiBMH2*]).ura3
bmhs-RiBMH2	MATa.his3Δ1.leu2Δ0.met15Δ0.ura3Δ0.BMH1::KanMX(Gal7[*RiBMH2*]).BMH2::ura3

Table S2. Primers used in this study

Primers	Sequences（5'-3'）	Primers	Sequences（5'-3'）
1433F	CGCTGAGCAAGCCGAACG	201ATGF3	GATTCCCCATGGTGACCTCTAATAAAAAGGT
1433R	AGTCCAAAGGGTCAAGTTATC T	201TAGR2	CTAATTATGGATCCTTTGATTCTTCAGCTTCG
201spF1	TGGGGAGGAGAAAGGAATGTTTGTTG	201F	TCACTGTGGAGGAACGTAATCT
P_{Gal7}F	CCCGAGCTCTTTGCCAGCTTACTATCCTTC	201spR2	AACAAACATTCCTTTCTCCTCCCCAT
P_{Gal7}R	CTCACTAGTTTTTGAGGGAATATTCAACTGTT	pMRI-12F1	CTTTATTCCGGCCTGGAGTCAAAAGAGGAAGCTCGGTGGCAA AT TTTGCCAGCTTACTATCCTTCTT
FmAct	CCTCACGCCATTCTTCGAC	pMRI-12R1	ACATATATAGATATAGATATATAATTGCAATAATGAACTACAA ATTATTACATGTTCTTTCCTGCGTTATCCCCTG
FmAct	CAGCGGTTTGCATTTCTTGT	pSH47F	GGGGGAAAAGCAAAGAACGAGAAAACTTGGACAGAAGGTTA ATACTCTAGATTGTACTGAGAGTGCAC
201F	TCACTGTGGAGGAACGTAATCT	pSH47R	CTGGAGTGGTAAATCTTCATTTCCCCTTGTATTTCTCAGCGCTC TTATTTGGTTGTGTGCGGTATTTCACACCGC
201R	TCAGCAATGGCATCATCAAA	BMH1upF	GCTTACTAGCCGGCTAGG
QT	CCAGTGAGCAGAGTGACGAGGACTCGAGCTC AAGCTTTTTTTTTTTTTTTTT	BMH1downR	GTGAAGATACAGGTGCGACAAAC
QC	CCAGTGAGCAGAGTGACGAGGACTCGAGCTC AAGCCCCCCCCCCCCCCCCC	BMH2upF	GAGATAATAATACAGCG
QO	CCAGTGAGCAGAGTGACG	BMH2downR	GAGGTGCGTATACAATGG
QI	GAGGACTCGAGCTCAAGC	201PR2	GGTCACCATGGTGTGAAAAAAAACGGAATAATTT
Ri14-3-3F	GCAAGCCGAACGTTATGATG	Fm201RTF	CGGGTGAAAGACGAAAAGAAGC
Ri14-3-3R	GGCAAGGATATCCGAGCATAC	Fm201RTR	CAAGTCCGAGACGAATAGGATGAGT
RiBMH2ATG	ATGGTCGAAGCTCAAACAC	UraF	AGGCGGCAGAAGAAGTAACA
RiBMH2TAA	TTAAGCTTCTGCTTCTTCCTTTG	UraR	AATGCGTCTCCCTTGTCATC
